# Importance of Will in Resilience and Recovery: Findings From the BLSA

**DOI:** 10.1093/geroni/igaa057.3032

**Published:** 2020-12-16

**Authors:** Eleanor Simonsick, Michael Griswold, B Gwen Windham

**Affiliations:** 1 National Institute on Aging, Bethesda, Maryland, United States; 2 The University of Mississippi Medical Center, Jackson, Mississippi, United States; 3 University of Mississippi Medical Center, Jackson, Mississippi, United States

## Abstract

Using data from 743 initially well-functioning men and women (49.5%) aged ≥60 in the Baltimore Longitudinal Study of Aging we interrogated the association between physical reserve operationalized as fast 400m walk performance scaled from 0 to 4 and psychological reserve (“will”) operationalized as personal mastery (high versus not) and likelihood of recovery from a decline of ≥2 points in reported walking ability. Of the 35% who declined 1-2 years post study baseline, 54% recovered 1-2 years later and 45% did not. Controlling for age, sex, race and initial walking ability, for each increment in reserve, likelihood of recovery was 43% greater (95% confidence interval (1.10, 1.85); p=.007). This association was most pronounced in women (odds ratio=1.84; 95% CI (1.19, 2.86; p=.006). Personal mastery showed no association with likelihood of recovery. Continuing work will further explore alternative operationalizations of “will”. Part of a symposium sponsored by Epidemiology of Aging Interest Group.

